# The 150 most important questions in cancer research and clinical oncology series: questions 6–14: Edited by *Chinese Journal of Cancer*

**DOI:** 10.1186/s40880-017-0200-0

**Published:** 2017-03-27

**Authors:** Chinese Journal of Cancer

**Affiliations:** Guangzhou, China

**Keywords:** Metastasis, Nasopharyngeal carcinoma, Colorectal cancer, Glioblastoma, High endothelial venules, Dynamic imaging, Pre-metastatic niche

## Abstract

To accelerate our endeavors to overcome cancer, *Chinese Journal of Cancer* has launched a program of publishing 150 most important questions in cancer research and clinical oncology. In this article, nine more questions are presented as followed. Question 6. Why do nasopharyngeal carcinomas rarely metastasize to the brain? Question 7. Can distant spread of cancer cells be blocked by inhibiting the remodeling of high endothelial venules in the sentinel lymph node? Question 8. What sort of live-imaging techniques can be developed to directly observe the dynamic processes of metastasis? Question 9. How does chronic hepatitis prevent liver metastasis from colorectal cancer? Question 10. How many types of host cells contribute to forming the pre-metastatic niche in the lung favorable for metastasis? Question 11. Why do cancers rarely metastasize to the small bowel? Question 12. Why do glioblastomas rarely metastasize outside the central nervous system? Question 13. Despite increased understanding of the molecular genetic events leading to the development and progression of high-grade gliomas, these tumors are the most therapeutically refractory among all human cancers. What then would be the most effective therapeutic approaches to treat what in essence can be regarded as a whole brain malignancy, since even a surgical resection of greater than 99% of tumor tissues is invariably associated with recurrence? Question 14. The blood–brain barrier (BBB) effectively limits a wide variety of potential therapeutic agents from reaching glioma cells widely dispersed in the brain. What therapeutic approaches can be used to breach the BBB and allow therapeutic agents to seek out and kill these tumor cells?

To accelerate our endeavors to overcome cancer, *Chinese Journal of Cancer* has launched a program of publishing 150 most important questions in cancer research and clinical oncology [1], with the first five questions been published in the last two issues [2, 3]. In this article, questions 6–14 are selected and presented. *Chinese Journal of Cancer* is still open to collect more key questions in cancer research and clinical oncology. Please send us your thoughtful questions to Ms. Ji Ruan via email: ruanji@sysucc.org.cn.

## Question 6: Why do nasopharyngeal carcinomas rarely metastasize to the brain?

### Background and implications

Of all head and neck cancers, nasopharyngeal carcinoma (NPC) has the highest metastasis rate. NPCs commonly metastasize to the lymph nodes, bone, liver, and lung. Given the close anatomic location of the nasopharynx to the brain, NPCs rarely metastasize to the brain. Determining the underlying molecular mechanism(s) may be very helpful for preventing brain metastases from other types of malignancies that are prone to spread to the brain.

#### Submitter

Chao-Nan (Miles) Qian.

#### Affiliation and email

Sun Yat-sen University Cancer Center, Guangzhou, Guangdong, P. R. China.

qianchn@sysucc.org.cn.

## Question 7: Can distant spread of cancer cells be blocked by inhibiting the remodeling of high endothelial venules in the sentinel lymph node?

### Background and implications

The sentinel lymph node is the most common first station in the metastatic cascade of carcinomas. The occurrence rate of distant metastasis is much higher in the patients with sentinel lymph node involvement than in those without sentinel lymph node metastasis. Therefore, the prognosis of cancer patients with sentinel lymph node involvement is usually poorer than that of the patients without sentinel lymph node involvement.

Our previous investigation has confirmed that primary tumor can induce vascular remodeling of high endothelial venules (HEVs) in the sentinel lymph node even before the arrival of metastatic cancer cells. The normal physiological function of HEVs is to provide an extravasation site for circulating naïve lymphocytes into the lymphoid tissue. Therefore, there are few or no red blood cells inside normal HEVs. The HEV remodeling induced by primary tumor is characterized by (1) enlargement of HEV lumen space; (2) reduced vessel wall thickness; (3) increased proliferation rate in the endothelial cells of HEV; and (4) increased red blood cell number inside the HEV lumen space. After cancer cell arrival, the metastatic cancer nests can unlimitedly expand and eventually occupy the whole lymph node, while the remodeled HEVs become the mother vessels of the metastatic tumor accompanied by further remodeling of HEVs characterized by the loss of their normal immunological function for lymphocyte extravasation.

It is critical to clarify whether HEV remodeling in the sentinel lymph node could facilitate further spread of cancer cells from the sentinel lymph node to distant organs. If our speculation of the role of HEV remodeling in cancer metastasis is true, we could then block further spread of cancer cells from the sentinel lymph node by inhibiting the remodeling procedure of HEV. Answering this question would open a new door for us to block tumor metastasis and to significantly enhance cancer patient survival rate.

#### Submitter

Chao-Nan (Miles) Qian.

#### Affiliation and email

Sun Yat-sen University Cancer Center, Guangzhou, Guangdong, P. R. China.

qianchn@sysucc.org.cn.

## Question 8: What sort of live-imaging techniques can be developed to directly observe the dynamic processes of metastasis?

### Background and implications

There is extensive work on investigating cancer metastasis in animal models, particularly end-point data on established metastasis, with standard textbook models commonly describing classical sequential models of cancer metastasis (“late metastasis”). In recent years, these models are challenged by early metastasis models, as more data accrue on clonality of distant metastasis. While live-imaging animal models allow monitoring of these processes in animals, there is little direct data and observations on the dynamic processes of cancer metastasis in humans, such as tumor cell intravasation, transport through circulation, and extravasation at distant site. It would be important to establish live-imaging techniques using innovative bioengineering approaches that would allow for sensitive detection and measurement of these processes. Such observations will certainly shed more light on the prevention of cancer metastasis.

#### Submitter

Min-Han Tan.

#### Affiliation and email

Institute of Bioengineering and Nanotechnology, Singapore, Singapore.

minhan.tan@gmail.com.

## Question 9: How does chronic hepatitis prevent liver metastasis from colorectal cancer?

### Background and implications

Liver metastasis has been commonly seen in over 50% of patients with colorectal cancer. Chronic hepatitis caused by hepatitis B and C virus infection has been recognized as precancerous alteration for hepatocellular carcinoma. Interestingly, in colorectal cancer patient with chronic hepatitis, liver metastasis is rare. Obviously, chronic hepatitis prevents the manifestation of liver metastasis from colorectal cancer. Determining the underlying molecular mechanism(s) may be very helpful for preventing liver metastases from other types of malignancy that are prone to spread to the liver.

#### Submitter

Zhongguo (Roy) Zhou.

#### Affiliation and email

Sun Yat-sen University Cancer Center, Guangzhou, Guangdong, P. R. China.

zhouzhg@sysucc.org.cn.

## Question 10: How many types of host cells contribute to forming the pre-metastatic niche in the lung favorable for metastasis?

### Background and implications

It is estimated that approximately 70% of cancer patients are died of tumor metastasis; among the patients who are succumbed to tumor metastasis, 20%–30% suffer from pulmonary metastasis. Numerous studies have demonstrated that forming the pre-metastatic niche in the lung is a critical step in the progression of pulmonary metastasis. Several types of primary tumor displayed the potential ability to “prepare” a supportive and receptive microenvironment in the lung for licensing circulating tumor cells (CTCs) colonization even before their arrival. Interestingly, compelling evidence showed that the formation of pre-metastatic niche was greatly associated with the interaction between tumor cells and host cells. In the stress of tumor-derived secreted factors and extracellular vesicles derived from the primary microenvironment, the local lung stroma can be reformed to be a favorable secondary microenvironment for settlement and progression of CTCs. The known cell types recruited to this altered microenvironment for metastasis include VEGFR1^+^ VLA-4^+^ bone marrow-derived cells, myeloid-derived suppressor cells, myeloid cells (Mac1^+^, CD11b^+^ CD68^+^ F4/80^+^, CD11b^+^/Mac1^+^, CD11b^+^/Gr-1^+^, CD11b^+^/Ly6C^med^/Ly6G^+^), monocytes (CD11b^+^ Ly6C^+^, IE2^+^, CD11b^+^/Gr1^+^), CD11b^+^ Ly6G^+^ Ly6C^+^ granulocytes, tumor-associated macrophages, regulatory T cells, VEGFR1^+^ VLA-4^+^ hematopoietic progenitor cells, endothelial progenitor cells, and neutrophils.

It is speculated that multiple cell types from the host are recruited to the pre-metastatic niche through a well-organized procedure coordinated by the primary tumor. Before unveiling the crosstalk among different host cell types, an essential question would be as follows: have we identified all cell types in the pre-metastatic niche? Answering this question would be very useful for better understanding the formation of pre-metastatic niche in the lung and other organs.

#### Submitter

Chunlin Ou.

#### Affiliation and email

Central South University Cancer Research Institute, Changsha, Hunan, P. R. China.

ouchunlin@csu.edu.cn.

## Question 11: Why do cancers rarely metastasize to the small bowel?

### Background and implications

Of all the organs that may have cancer metastasis, the small bowel has the lowest metastasis rate. Determining the underlying molecular mechanism(s) through which the small bowel keeps away from metastasis may be very helpful for preventing metastases from different malignancies that are prone to spread to specific organs.

#### Submitters

Xiaowen Wang, Dave Hoon.

#### Affiliation and email

Translational Molecular Medicine, John Wayne Cancer Institute, CA, USA.

xiaowen.wang@providence.org.

## Question 12: Why do glioblastomas rarely metastasize outside the central nervous system?

### Background and implication

Contrasting to extracranial cancers, glioblastomas are primarily formed in the brain and rarely metastasize outside the central nervous system even after neurosurgery which can destroy the blood–brain barrier. Actually, in rare circumstances, glioblastoma cells can spread to the lung, liver, bone, lymph node, and other distance organs. In animal experiments, glioblastoma cells can grow subcutaneously in immuno-deficient mice. All these facts suggest that the brain microenvironment is not an indispensable factor for the growth of glioblastoma cells. Other key limiting factors should exist to prevent the spreading or survival of glioblastoma cells outside the central nervous system. Determining these factors may be very helpful for understanding the mechanism of cancer metastases.

#### Submitter

Yong-Gao Mou.

#### Affiliation and email

Sun Yat-sen University Cancer Center, Guangzhou, China.

mouyg@sysucc.org.cn.

## Question 13: Despite increased understanding of the molecular genetic events leading to the development and progression of high-grade gliomas, these tumors are the most therapeutically refractory among all human cancers. What then would be the most effective therapeutic approaches to treat what in essence can be regarded as a whole brain malignancy, since even a surgical resection of greater than 99% of tumor tissues is invariably associated with recurrence?

### Background and implications

The greatest single advance in the treatment of high-grade gliomas, and more specifically, glioblastomas (GBM), over the past 30 years has been the addition of temozolomide (TMZ) to the postoperative radiotherapy. This has resulted in only a 2.5-month increase in the median overall survival, to 14.5 months for patients receiving combination therapy, compared with 12.1 months for those receiving radiotherapy alone. Clearly, new therapeutic approaches are needed to treat patients with GBMs.

What might these new therapeutic approaches consist of? First, it probably will entail a combination of new targeted chemotherapeutic agents that will attack molecular checkpoints that drive GBMs to proliferate. Second, it might include immunotherapeutic approaches directed against molecular targets such as programmed death-1 (PD-1) and tumor-specific antigens expressed on GBM cells. Third, it might include the use of oncolytic viruses such as genetically engineered herpes and adenoviruses that will be able to selectively target infiltrating tumor cells interspersed in so-called “normal” brain. Progress is being made with each of these approaches, but the challenge will be to effectively combine them with the current standard treatment to eradicate residual tumor cells that, up until this time, have evaded current chemoradiotherapeutic approaches.

#### Submitter

Rolf F. Barth.

#### Affiliation and email

The Ohio State University, Columbus, OH, USA.

rolf.barth@osumc.edu.

## Question 14: The blood–brain barrier (BBB) effectively limits a wide variety of potential therapeutic agents from reaching glioma cells widely dispersed in the brain. What therapeutic approaches can be used to breach the BBB and allow therapeutic agents to seek out and kill these tumor cells?

### Background and implications

Curative treatment of glioblastomas requires effective delivery of a variety of therapeutic agents to glioma cells infiltrating the normal brain tissue. The BBB constitutes a significant barrier for the delivery of therapeutic agents with molecular weights greater than 200, making drug delivery to the brain more complex than to any other organ.

At the present time, a variety of approaches have been used to allow high molecular weight agents to traverse the BBB. These have included BBB disruption by ultrasound and hyperosmotic mannitol, the use of transporter carriers such as insulin-like growth factor-1 and novel brain-penetrating peptides such as ANG4043 that can be linked to therapeutic agents such as drug bioconjugates, and the direct intracerebral administration of therapeutic agents to the site of infiltrating glioma cells, completely bypassing the BBB. Each of these approaches are currently being evaluated, and hopefully one or more of them, combined with new therapeutic agents as described in the previous question, will be able to selectively target residual proliferating tumor cells that otherwise lead to tumor recurrence and death of the patient.

#### Submitter

Rolf F. Barth.

#### Affiliation and email

The Ohio State University, Columbus, OH, USA.

rolf.barth@osumc.edu.
